# Wnt5a promotes renal tubular inflammation in diabetic nephropathy by binding to CD146 through noncanonical Wnt signaling

**DOI:** 10.1038/s41419-020-03377-x

**Published:** 2021-01-18

**Authors:** Xiaomei Li, Jiejun Wen, Yang Dong, Qunzi Zhang, Jian Guan, Feng Liu, Ting Zhou, Ze Li, Ying Fan, Niansong Wang

**Affiliations:** 1 Department of Nephrology, Shanghai Jiao Tong University Affiliated Sixth People’s Hospital, Shanghai, China; 2 Therapy Center for Obstructive Sleep Apnea, Department of Otolaryngology, Shanghai Jiao Tong University Affiliated Sixth People’s Hospital, Shanghai, China; 3 Otolaryngology Institute of Shanghai Jiao Tong University, Shanghai, China; 4 Shanghai Key Laboratory of Sleep Disordered Breathing, Shanghai, China

**Keywords:** Kidney diseases, Inflammation

## Abstract

Immune and inflammatory factors have emerged as key pathophysiological mechanisms in the progression of diabetic renal injury. Noncanonical Wnt5a signaling plays an essential role in obesity- or diabetes-induced metabolic dysfunction and inflammation, but its explicit molecular mechanisms and biological function in diabetic nephropathy (DN) remain unknown. In this study, we found that the expression of Wnt5a and CD146 in the kidney and the level of soluble form of CD146 (sCD146) in serum and urine samples were upregulated in DN patients compared to controls, and this alteration was correlated with the inflammatory process and progression of renal impairment. Blocking the activation of Wnt5a signaling with the Wnt5a antagonist Box5 prevented JNK phosphorylation and high glucose-induced inflammatory responses in db/db mice and high glucose-treated HK-2 cells. Similar effects were observed by silencing Wnt5a with small-interfering RNA (siRNA) in cultured HK-2 cells. Knockdown of CD146 blocked Wnt5a-induced expression of proinflammatory cytokines and activation of JNK, which suggests that CD146 is essential for the activation of the Wnt5a pathway. Finally, we confirmed that Wnt5a directly interacted with CD146 to activate noncanonical Wnt signaling in HK-2 cells. Taken together, our findings suggest that by directly binding to CD146, Wnt5a-induced noncanonical signaling is a contributing mechanism for renal tubular inflammation in diabetic nephropathy. The concentration of sCD146 in serum and urine could be a potential biomarker to predict renal outcomes in DN patients.

## Introduction

Due to the increasing prevalence of diabetes, diabetic nephropathy (DN), as one of the most prevalent microvascular complications, has become the most common cause of end-stage renal disease (ESRD) worldwide^1–3^. Clear evidence indicates that the pathogenesis of DN is multifactorial, with hyperglycemia, hemodynamic changes, oxidative stress, advanced glycation end products and so on^4–6^. Although DN is considered a nonimmune disease, emerging evidence suggests that inflammation has emerged as a key pathophysiological mechanism of DN^7,8^. Through the regulation of proinflammatory cytokines, chemokines, and signaling pathways, the inflammatory process plays an important role in the progression of renal injury^9^. Therefore, investigations into the inflammatory mechanisms involved in the development of diabetic kidney injury may provide new potential targets for anti-inflammatory therapeutic strategies against DN.

Wnt5a, a noncanonical member of the Wnt family glycoproteins, is a representative ligand of noncanonical Wnt signaling^10,11^. Wnt5a plays important roles in development, cell proliferation, and cell migration^12,13^. Emerging evidence indicates that Wnt5a is a novel inflammatory mediator in metabolic diseases^14,15^. In animal models of obesity and type 2 diabetes, Wnt5a triggers the activation of signaling pathways through JNK (c-jun N-terminal kinase) activation, and then contributes to low-grade inflammation, insulin resistance, and metabolic dysfunction in adipose tissue and atherosclerotic plaque^16–19^. Furthermore, the serum concentration of Wnt5a is significantly increased in severely obese patients (BMI > 40 kg/m^2^) and is positively correlated with atherosclerosis and cardiovascular risk^15,20,21^. Although increasing evidence suggests the role of Wnt5a in metabolic inflammation, little is known about the role of noncanonical Wnt5a signaling in the development of renal injury in diabetic patients.

CD146 is a cell adhesion molecule that belongs to a subgroup of the immunoglobulin (Ig) superfamily. It plays a fundamental role in cell signaling, migration, proliferation, activation, and inflammatory reactions^22,23^. Our previous work revealed that high glucose (40 mM) induced the expression of CD146 in human proximal tubular epithelial (HK-2) cells^24^. In addition, the expression of CD146 in the renal tubules and the plasma concentration of the soluble form of CD146 (sCD146) were upregulated in DN patients^25^. Another study indicated that CD146 could interact with Wnt5a and act as a Wnt5a receptor to activate noncanonical Wnt signaling via a Wnt/Dvl/JNK cascade in zebrafish^26^. However, the explicit molecular mechanisms of CD146 and noncanonical Wnt5a signaling in DN still need to be fully explored.

In the current study, we combined human, mouse, and cellular studies to provide evidence that CD146 directly binds to Wnt5a, activates noncanonical Wnt signaling, and promotes subsequent inflammatory responses in diabetic nephropathy. In addition, by examining the levels of sCD146 in serum and urine samples, we further verified whether sCD146 can be used as a new biomarker for evaluating kidney damage in diabetic patients.

## Methods

### Human subjects

The human study was approved by the Ethics Committee of Shanghai Jiao Tong University Affiliated Sixth People’s Hospital and conformed to the principles of the Declaration of Helsinki. Written informed consent was obtained from every enrolled individual prior to blood sampling and kidney biopsy. Renal biopsy tissues, serum, and urine samples were obtained from 15 patients with DN, 5 patients with minimal change disease (MCD), and 5 normal control nephrectomy samples. In the DN group, all patients were excluded due to the coexistence of other types of kidney disease. The pathologic classification of DN was evaluated with Tervaert’s classification by at least two renal pathologists^27^. The clinical and pathological data of DN patients were shown in Supplementary Table S3. Nondiabetic nephrotic subjects with MCD were included to confirm that Wnt5a overexpression is specific to DN but not secondary to proteinuria.

### Animal models

Six-week-old male db/db (C57BLKS/J-*lepr*^*db*^/*lepr*^*db*^) mice and their nondiabetic wild-type littermates (WT) were provided by the Model Animal Research Center of Nanjing University (Nanjing, China). The animals were acclimatized to the laboratory before use. At 8 weeks of age, mice were classified into four groups (*n* = 8 each): WT + Box5 and db/db+Box5 mice were i.p. injected with the Wnt5a inhibitor, Box5 (1 mg/kg body weight), every other day for 12 weeks from 8 weeks of age; WT + Vehicle and db/db+Vehicle mice received 0.9% physiologic saline on the same schedule as the control mice.

Blood glucose levels and body weight were measured every 2 weeks. Blood glucose levels were determined using a glucose meter (Roche, Basel, Switzerland). Twenty-four-hour urine was collected from mice using metabolic cages every 2 weeks. Urinary albumin and creatinine levels were measured using enzyme-linked immunosorbent assay (ELISA) kits (Abcam, Cambridge, USA). The urine albumin excretion rate was expressed as the ratio of albumin to creatinine. All mice were sacrificed at 20 weeks and blood and tissue samples were harvested. Serum creatinine levels were determined using the QuantiChrom Aassay Kit (BioAssay Systems, Hayward, CA, USA). The animal experiments were approved by the Laboratory Animals Ethical Committee of Shanghai Jiao Tong University Affiliated Sixth People’s Hospital.

### Measurement of serum and urine sCD146

The concentration of sCD146 in human serum and urine samples was determined with a commercial enzyme-linked immunosorbent assay (CY-QUANT ELISA, BioCytex, Marseille, France) according to the manufacturer’s instructions. The sCD146 concentration in mouse serum was measured using a mouse CD146 ELISA kit (Raybiotech, Norcross, GA, USA).

### Kidney histologic analysis and immunostaining analysis

Paraformaldehyde-fixed kidney samples were embedded in paraffin or frozen in OCT compound and then sectioned to 4-μm thickness for light or fluorescence microscopy. Hematoxylin-eosin (HE), periodic acid-Schiff (PAS), and Masson trichrome staining were performed to assess histological injury. Tubular injury was defined as tubular atrophy and thickening of the tubular basement membrane. Interstitial lesions were defined as interstitial fibrosis and infiltration of inflammatory cells. Tubulointerstitial injury was scored semiquantitatively by two independent observers in a blinded manner, using the following scoring system as described previously^26^: 0: no tubular injury; 1: <10% of tubules injured; 2: 10% to <25% of tubules injured; 3: 25 to <50% of tubules injured; 4: 50% to <75% of tubules injured; 5: ≥75% of tubules injured.

Immunostaining was performed as described previously^26^. For human biopsy samples, kidney sections were stained using the following antibodies: anti-Wnt5a (MA5-15502, Thermo Fisher Scientific), anti-CD146 (MA5-29413, Thermo Fisher Scientific), anti-CD3 (A045201, Dako), anti-CD20 (M0755, Dako), anti-CD68 (IR60961, Dako), and anti-CCL-2 (MA5-17040, Thermo Fisher Scientific). The primary antibodies used for mouse tissue were as follows: anti-Wnt5a (PA5-95181, Thermo Fisher Scientific), anti-CD146 (ab75769, Abcam), anti-collagen-I (ab34710, Abcam), anti-CCL-2 (ab25124, Abcam), and anti-F4/80 (#30325S, Cell Signaling). The positive staining was qualified using Image-Pro Plus Software 6.0 (Media Cybernetics, Silver Spring, MD) and expressed as integrated optical density (IOD). The numbers of CD3^+^, CD20^+^, CD68^+^, and F4/80^+^ cells in the tubulointerstitium were counted in at least 20 equivalent high-power cortical fields (HPF ×400) and were expressed as the average number of cells per HPF.

### Cell culture and treatment

A human proximal tubular epithelial cell line (HK-2) was kindly provided by Dr. John Cijiang He (Icahn School of Medicine at Mount Sinai, New York, USA). The cells were cultured in DMEM/F12 (Sigma-Aldrich) medium supplemented with 10% fetal bovine serum (Gibco, Grand Island, NY), 1% penicillin, and streptomycin in an atmosphere of 5% CO_2_ and 95% air at 37 °C.

In all experiments, the cells were starved in a serum-free medium for 12–16 h, and then stimulated with 30 mmol/L of high glucose (HG, Sigma-Aldrich) for 24 h. Box5 (100 μM/L) or recombinant Wnt5a protein (200 ng/mL) was given for the indicated time periods. The cells were pretreated with Box5 2 h before the glucose treatment.

For gene disruption, HK-2 cells were plated in six-well plates and transiently transfected with 50 nM Wnt5a-specific small-interfering RNA (siRNA) (Sigma-Aldrich, St. Louis, MO, USA) or 1 μg CD146-specific siRNA (Thermo Fisher Scientific, Waltham, MA, USA) using Lipofectamine 3000 (Invitrogen, Grand Island, NY) according to the manufacturer’s protocol. The efficacy of knockdown was determined by real-time PCR and western blotting (Supplementary Fig. S2). After transfection for 48 h, these cells were treated with HG for another 24 h. Cell supernatants, lysates, and RNA were collected for subsequent experiments.

### Measurement of proinflammatory cytokines

TNF-α, IL-6, and CCL-2 protein levels in culture supernatants were determined by commercial ELISA kits (Arigo Biolaboratories, Taiwan) according to the manufacturer’s instructions.

### Real-time PCR

Total RNA was extracted from the renal cortex sample or cultured HK-2 cells using TRIzol reagent (Invitrogen, Carlsbad, CA). RNA was reverse-transcribed using the PrimeScript™ RT Reagent Kit with gDNA Eraser (Takara, Kyoto, Japan). Real-time PCR was performed using SYBR Green Master Mix (Qiagen) with an Applied Biosystems Step One Plus Real-time PCR system (Applied Biosystems, Foster City, CA, USA). Primers used for quantitative polymerase chain reaction are shown in Supplementary Table S1. Polymerase chain reaction product specificity was confirmed by melting curve analysis. Relative quantification of gene expression was carried out using the 2^−ΔΔCT^ method, and all samples were assessed in triplicate. Gene expression was normalized to that of GAPDH, which was assessed in separate tubes to permit quantification of the target gene.

### Western blot analysis

Tissue or cells were lysed with RIPA buffer containing protease and phosphatase inhibitor cocktail. After protein concentration determination, cell lysates were subjected to western blot analysis using specific antibodies shown in Supplementary Table S2. We repeated each western blot analysis using protein from three different and separate experiments. The specific protein bands were scanned using a western blotting detection system (BIO-RAD).

### Coimmunoprecipitation (Co-IP)

One microgram of V5-tagged Wnt5a and 1 μg of FLAG-tagged CD146 or 2 μg of each plasmid were transfected into HK-2 cells for 48 h. Cells were lysed with RIPA buffer and incubated with anti-V5 antibody (GenScript) or anti-FLAG antibody (Cell Signaling) for overnight at 4 °C and the precipitated materials were used for western blot analysis using anti-FLAG and anti-V5 antibodies. For co-IP with endogenous protein, HK-2 cells were stimulated with HG for 24 h with or without pretreatment with Box5 (100 μM/L). Cells were then lysed as described above and incubated with anti-wnt5a or anti-CD146 antibodies for IP, followed by western blot analysis with anti-CD146 or anti-wnt5a antibodies.

### Statistical analysis

All data are shown as the mean value ± standard deviation (SD). All experiments were performed in triplicate. Significant differences were determined with unpaired two-tailed *t*-test or one-way ANOVA followed by Bonferroni multiple comparison test using Prism (GraphPad Software). Statistical significance was set at *P* < 0.05, *P* < 0.01, and *P* < 0.001.

## Results

### The expression levels of Wnt5a and CD146 were upregulated in DN patients

**Fig. 1:**
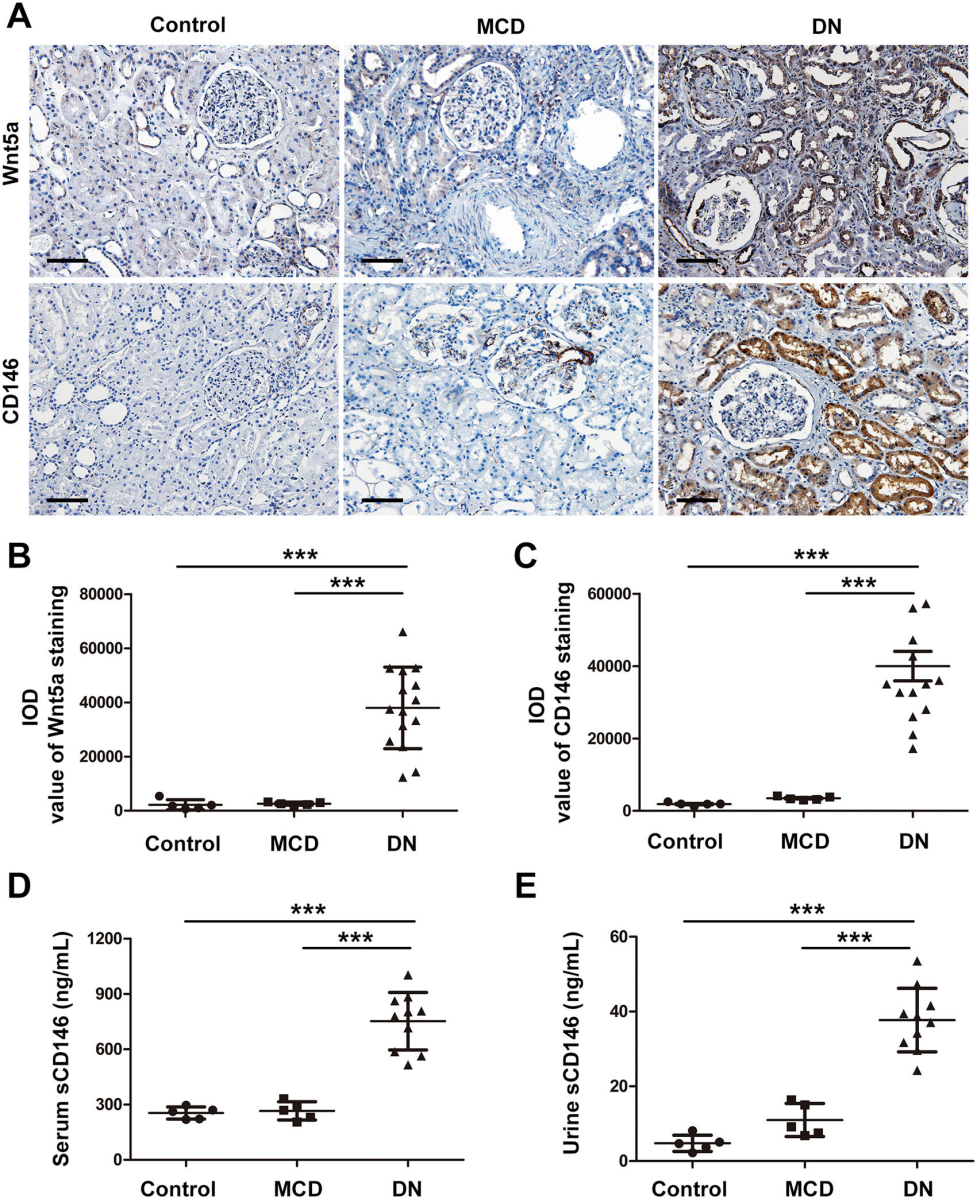
The expression levels of Wnt5a and CD146 were upregulated in diabetic neuropathy (DN) patients. **A** Representative immunostaining of Wnt5a and CD146 on normal kidney sections of nephrectomy samples (*n* = 5) and kidney biopsy samples of patients with minimal change disease (MCD) (*n* = 5) and DN (*n* = 15). Original magnification, ×200; scale bar, 100 μm. **B** Quantitative analysis of Wnt5a staining in kidney sections from each group according to the average integrated optical density (IOD). ***P < 0.001. **C** Quantitative analysis of expression of CD146 staining on kidney sections of each group by the average IOD. ***P < 0.001. **D**, **E** The concentration of soluble CD146 (sCD146) in serum and urine samples of each group. ***P < 0.001. Immunostaining was performed in duplicate. Significant differences were determined by unpaired two-tailed *t*-tests.

To examine whether Wnt5a signaling was involved in DN, we performed Wnt5a immunostaining on kidney biopsy samples of patients with DN and minimal change disease (MCD) and on normal kidney sections of nephrectomy samples. We confirmed that the expression of Wnt5a detected by staining, was significantly increased in the kidneys of DN patients compared with MCD and normal control kidneys, especially in the tubular compartment (Fig. 1A, B). Since CD146 is identified as a noncanonical Wnt5a receptor^26^, we checked the expression of CD146 in the kidneys of these patients. CD146 was significantly increased in both the glomeruli and arteries in patients with DN, while the increase was much stronger in the renal tubules (Fig. 1A, C). In addition, the sCD146 level in serum and urine samples was much higher in DN patients than that in the MCD and normal control groups (Fig. 1D, E). Therefore, we focused on the role of Wnt5a and CD146 in the development of DN in subsequent studies.

### The expression of Wnt5a and CD146 correlated with the inflammatory process and progression of renal impairment in DN patients

**Fig. 2:**
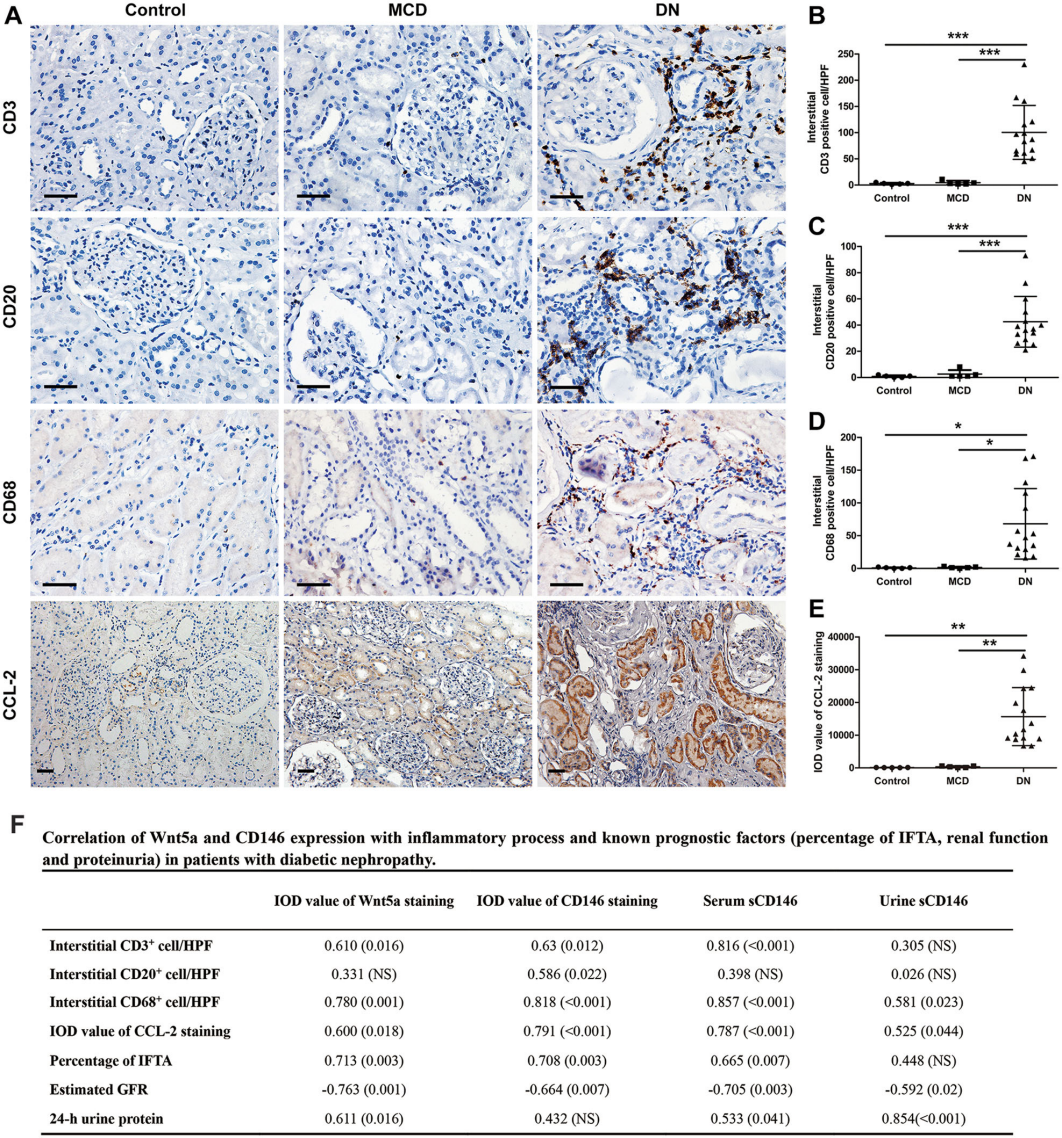
Assessment of renal inflammation in human kidney biopsies. **A** Immunostaining using specific antibodies for different immune cells, including T lymphocytes (CD3^+^ cells), B lymphocytes (CD20^+^ cells) and monocytes/macrophages (CD68^+^ cells), was performed on normal subjects, MCD patients, and DN patients. Original magnification, ×400; scale bar, 50 μm. Immunohistochemical staining of the proinflammatory marker, chemokine (C-C motif) ligand 2 (CCL-2) was also performed. Original magnification, ×200; scale bar, 50 μm. **B**–**D** The numbers of CD3^+^, CD20^+^, and CD68^+^ cells per high-power field (HPF) were counted in the tubulointerstitium. ***P < 0.001, *P < 0.05. **E** Quantitative analysis of the expression of CCL-2 staining on kidney sections of each group according to the average IOD. **P < 0.01. Immunostaining was performed in duplicate. Significant differences were determined by unpaired two-tailed *t*-tests. **F** Correlation of Wnt5a and CD146 expression with inflammatory process and known prognostic factors (percentage of IFTA, renal function, and proteinuria) in patients with diabetic nephropathy. Data are *r* value based on Pearson and Spearman correlation analysis; (*P* value); NS is not significant. IFTA, interstitial fibrosis and tubular atrophy; Estimated GFR, Estimated glomerular filtration rate; HbA1c, hemoglobin A1c.

To further examine whether Wnt5a signaling played a role in the inflammatory process of DN, immunohistochemistry studies were performed with specific markers to characterize cellular infiltration. Different immune cells, including T lymphocytes (CD3^+^ cells), B lymphocytes (CD20^+^ cells), and monocytes/macrophages (CD68^+^ cells) were observed in the kidneys of DN patients, whereas few inflammatory cells were detected in MCD patients and normal control subjects (Fig. 2A–C). Next, immunohistochemical staining of chemokine (C-C motif) ligand 2/monocyte chemoattractant protein-1 (CCL-2/MCP-1), a key chemokine involved in the recruitment of inflammatory cells in the kidney, was performed. We found that CCL-2 staining was much more pronounced in DN kidney sections than in MCD and normal kidney sections (Fig. 2A, D).

Quantitative analysis on kidney sections demonstrated that the IOD value of Wnt5a and CD146 staining was positively correlated with the number of interstitial CD3^+^ lymphocytes, CD68^+^ monocytes/macrophages, and the IOD value of CCL-2 staining. In addition, the IOD values of Wnt5a and CD146 were correlated with known predictors of DN progression, such as the percentage of interstitial fibrosis and tubular atrophy (IFTA), eGFR, and 24-h proteinuria. The concentration of sCD146 in serum and urine samples was also related to some of the tubulointerstitial inflammatory indicators and known prognostic factors in DN patients (Fig. 2F). However, no correlation was found between Wnt5a and CD146 expression and blood glucose and hemoglobin A1c (HbA1c) in all subjects with DN (Supplementary Fig. S2). Taken together, these data suggest that the expression of Wnt5a and CD146 is upregulated and correlates with the inflammatory process and the progression of DN.

### Wnt5a antagonist box5 alleviated renal injury in db/db mice

**Fig. 3:**
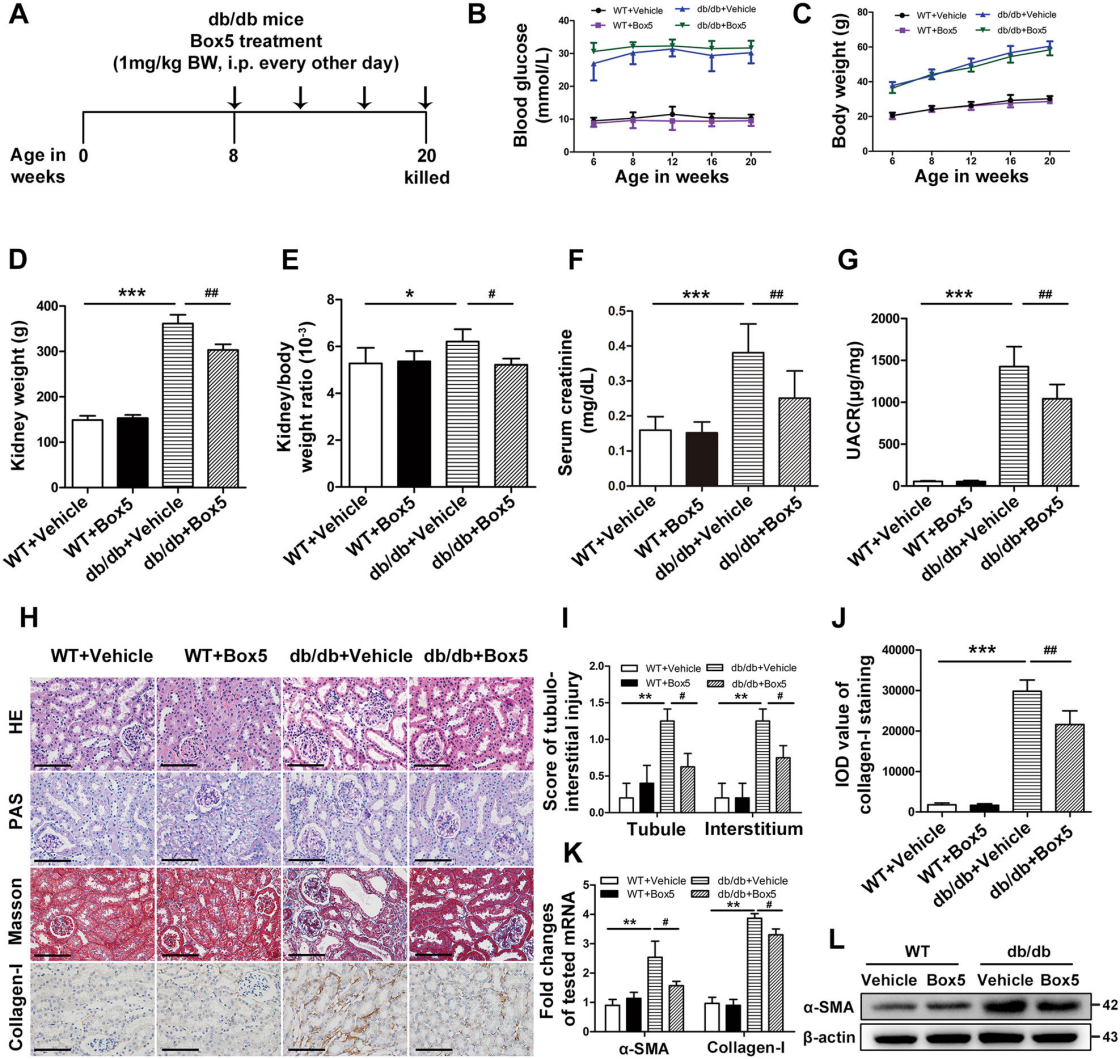
Wnt5a antagonist box5 alleviated renal injury in db/db mice. **A** The treatment protocol of a Wnt5a competitive antagonist, Box5, for db/db mice (*n* = 8). Arrows indicate when mice received i.p. injections of vehicle or Box5 (1 mg/kg BW) for 12 weeks and then were killed at 20 weeks for study. **B**, **C** Blood glucose levels and body weight were measured every 2 weeks. **D**–**G** The kidney weight, ratio of kidney weight to body weight, serum creatinine and urine albumin-to-creatinine ratio (UACR) in mice from the WT + Vehicle, WT + Box5, db/db+Vehicle, and db/db+Box5 groups. ***P < 0.001, **P < 0.01, versus WT + Vehicle mice, ^##^*P* < 0.01, ^#^*P* < 0.05, versus db/db+Vehicle. **H** Renal histology evaluations of different groups were performed with hematoxylin and eosin (HE) and periodic acid-Schiff (PAS) staining. Collagen fiber deposition was determined with Masson trichrome staining and immunohistochemical staining of collagen-I. Original magnification, ×400; scale bar, 50 μm. **I** Semiquantitative analysis of tubulointerstitial injury in the kidneys of all groups. **P < 0.01, versus WT + Vehicle mice, ^#^*P* < 0.05, versus db/db+Vehicle. **J** Quantitative analysis of collagen-I staining in kidney sections of each group according to the average IOD. ***P < 0.001, versus WT + Vehicle mice, ^##^*P* < 0.01, versus db/db+Vehicle. Western blotting (**K**) and qPCR (**L**) were performed to analyze the expression of the profibrotic molecules collagen-I and alpha-smooth muscle actin (α-SMA) in the kidneys of these mice. **P < 0.01, versus WT + Vehicle mice, ^#^*P* < 0.05, versus db/db+Vehicle. Western blotting and immunostaining were performed in duplicate. Representative pictures of eight mice were shown. All values were presented as the mean ± SD. Two-sided unpaired *t*-tests and one-way ANOVA followed by Bonferroni multiple comparison test were used.

We next evaluated the potential protective effects of inhibiting the Wnt5a signaling pathway on kidney injury in db/db mice using a treatment protocol in which Box5, an antagonist of Wnt5a, was administered by i.p. injection (1 mg/kg body weight) to db/db mice for 12 weeks (Fig. 3A). The db/db mice had significantly increased blood glucose and body weight compared with the wild-type littermates (WT) (Fig. 3B, C). Though Box5 treatment did not affect the blood glucose and body weight levels of db/db mice, mice in the db/db+Box5 group had significantly lower kidney weight and ratio of kidney to body weight (KW/BW) than those in db/db+Vehicle controls (Fig. 3D, E). In addition, the mean serum creatinine and urine albumin-to-creatinine ratio (UACR) levels of the db/db group were much higher than those of the two WT groups, while this increase was significantly attenuated in the db/db+Box5 group (Fig. 3F, G). There was no obvious difference in any of the parameters between WT + Vehicle and WT + Box5 mice.

**Fig. 4:**
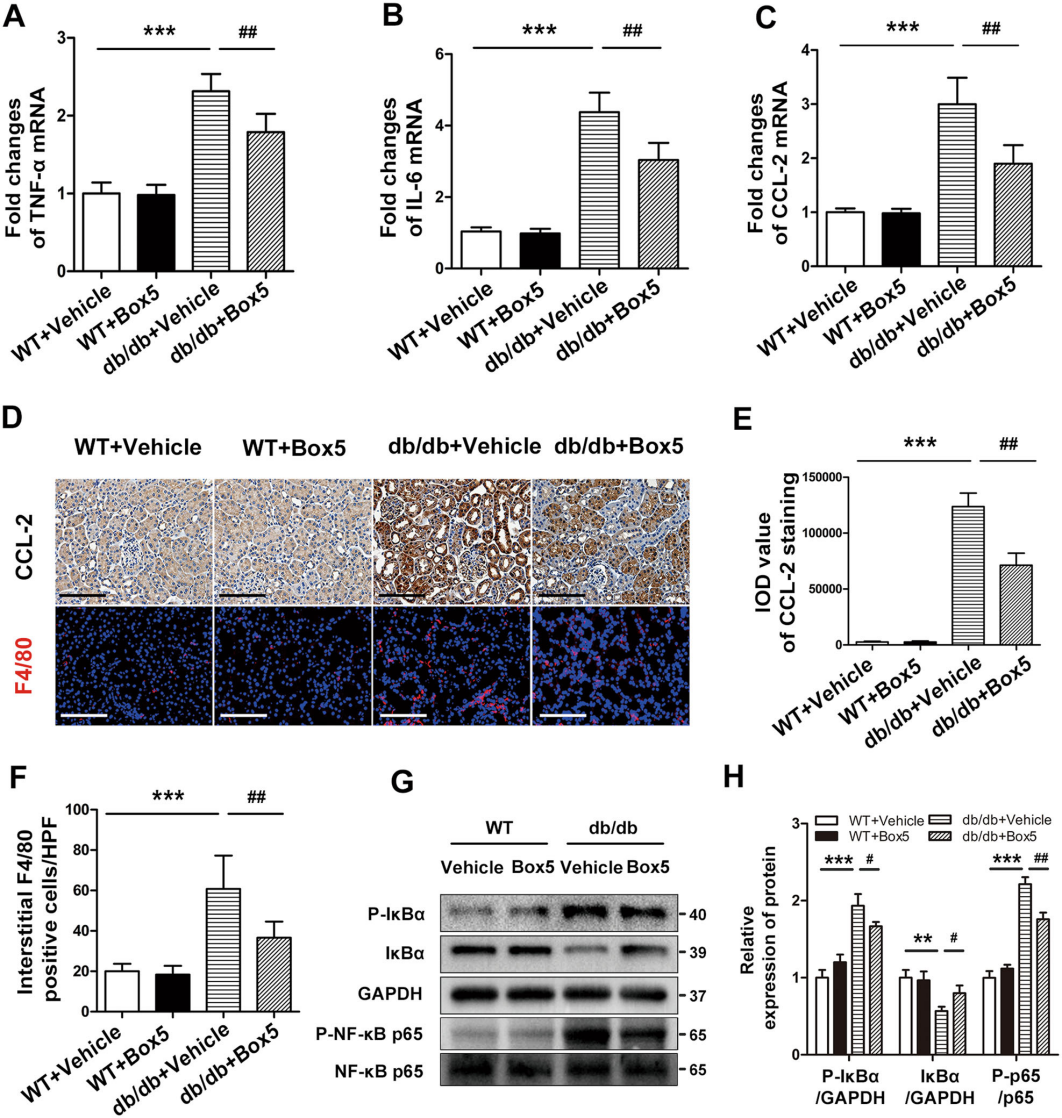
Box5 treatment prevented diabetes-induced renal inflammation in db/db mice. **A**–**C** The mRNA levels of the proinflammatory cytokines tumor necrosis factor-a (TNF-α), interleukin (IL)-6, and CCL-2/MCP-1 were determined by real-time PCR in all groups. ***P < 0.001, versus WT + Vehicle mice, ^##^*P* < 0.01, versus db/db+Vehicle mice. **D** Immunohistochemical staining of proinflammatory cytokine CCL-2 in kidney tissue isolated from all groups. Renal macrophage infiltration in these groups was also evaluated by immunofluorescent detection of F4/80 (red dots). Original magnification, ×400; scale bar, 50 μm. **E** Quantitative analysis of CCL-2 staining in kidney sections according to each group by the average IOD. ***P < 0.001, versus WT + Vehicle mice, ^##^*P* < 0.01, versus db/db+Vehicle mice. **F** The numbers of F4/80^+^ cells per high-power field (HPF) were counted in the tubulointerstitium. ***P < 0.001, versus WT + Vehicle mice, ^##^*P* < 0.01, versus db/db+Vehicle mice. **G** Representative western blot analysis of phosphorylated IκB, IκB, phosphorylated NF-κB p65 and NF-kB p65 in renal tissue. **H** Quantification of western blots by densitometric analysis. ***P < 0.001, **P < 0.01 versus WT + Vehicle mice, ^##^*P* < 0.01, ^#^*P* < 0.05, versus db/db+Vehicle mice. PCR experiments were performed in triplicate. Western blotting and immunostaining were performed in duplicate. Representative pictures of eight mice were shown. All values were presented as mean ± SD. Two-sided unpaired *t*-tests and one-way ANOVA followed by Bonferroni multiple comparison test were used.

Hematoxylin and eosin (HE) and PAS staining revealed that except for diabetes-induced glomerular changes, the db/db mice had notable inflammatory cell infiltration, whereas the interstitial infiltrates were markedly reduced in the db/db+Box5 group compared with the db/db+Vehicle controls (Fig. 3H, I). Masson trichrome and type I collagen (collagen-I) staining revealed significant increases in IFTA in db/db mice, whereas these renal changes were alleviated in the Box5-treated db/db group (Fig. 4H–J). Consistent with our histologic findings, the mRNA expression levels of the profibrotic molecules collagen-I and alpha-smooth muscle actin (α-SMA) were significantly elevated in the kidneys of db/db mice compared with WT mice; however, Box5 treatment prevented these increases (Fig. 3K). In addition, Box5 treatment-induced parallel decreases in the protein expression of α-SMA in renal tissue (Fig. 3L).

In summary, these data suggest that the Wnt5a antagonist Box5 could alleviate kidney injury in db/db mice and ameliorate the progression of DN via a mechanism independent of insulin sensitizing effects.

### Box5 treatment prevented diabetes-induced renal inflammation in db/db mice

We next investigated whether the protective action of Box5 against DN is attributed to its anti-inflammatory capability. Our findings indicated that the mRNA levels of tumor necrosis factor-a (TNF-α), CCL-2, and interleukin (IL)-6 were significantly elevated in the kidney tissue of db/db mice, whereas Box5 treatment markedly reduced the levels of these proinflammatory cytokines (Fig. 4A–C). Immunohistochemical staining of CCL-2 also indicated that Box5 effectively prevented the increase in CCL-2 in the renal tissue of db/db mice (Fig. 4D, E). Moreover, the percentage of F4/80^+^ macrophage infiltration was markedly reduced in the tubulointerstitium of the db/db+Box5 group compared with the db/db+Vehicle control group (Fig. 4D, F).

We then determined the regulatory effect of Box5 on the phosphorylation of the IκB and NF-κB p65 subunits. Compared with those in the WT control, the phosphorylated IκB and NF-κB p65 protein levels were increased in the renal tissue of db/db mice, whereas Box5 administration markedly reduced the increase (Fig. 4G, H). Thus, these results indicate that the antagonist of Wnt5a significantly attenuates tubulointerstitial inflammation in db/db mice.

### Box5 prevented inflammatory responses by blocking the activation of noncanonical Wnt5a signaling

**Fig. 5:**
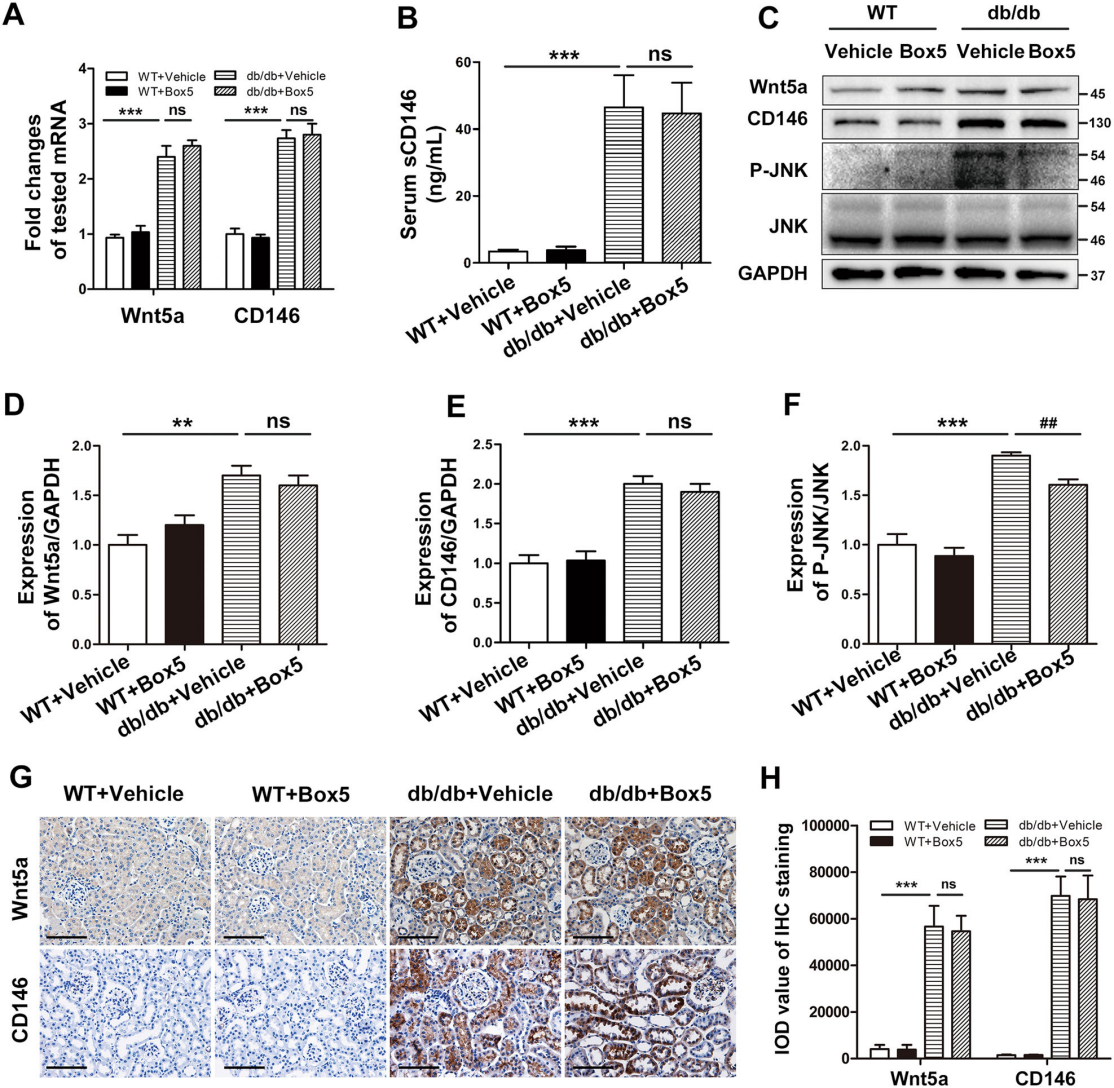
Box5 prevented inflammatory responses by blocking the activation of noncanonical Wnt5a signaling. **A** The mRNA levels of Wnt5a and CD146 were determined by real-time PCR in all groups. ***P < 0.001, versus WT + Vehicle mice, ns, no significance. **B** The concentration of sCD146 in the serum was determined by ELISA in all groups. ***P < 0.001, versus WT + Vehicle mice, ns, no significance. **C**–**F** Western blot analysis and densitometric quantification of Wnt5a, CD146, the phosphorylated JNK and total JNK in all groups of mice. ***P < 0.001, **P < 0.01 versus WT + Vehicle mice, ^##^*P* < 0.01, versus db/db+Vehicle mice, ns, no significance. **G** Immunohistochemical staining of Wnt5a and CD146 in kidney tissue of all groups. Original magnification, ×400; scale bar, 50 μm. **H** Quantitative analysis of the expression of Wnt5a and CD146 staining on kidney sections of each group according to the average IOD. ***P < 0.001, versus WT + Vehicle mice, ns, no significance. PCR experiments were performed in triplicate. Western blotting and immunostaining were performed in duplicate. Representative pictures of eight mice were shown. All values were presented as the mean ± SD. Two-sided unpaired *t*-tests and one-way ANOVA followed by Bonferroni multiple comparison test were used.

We next examined the expression of Wnt5a and CD146 in db/db mice. Real-time PCR, western blot analysis, and immunohistochemistry staining of renal tissue showed that the expression of Wnt5a and CD146 was significantly higher in the kidneys of db/db mice than in those of nondiabetic control mice (WT), whereas no significant difference was noted in the expression levels of Wnt5a and CD146 after Box5 treatment (Fig. 5A, C–G). Consistent with the observation in DN patients, immunostaining of Wnt5a and CD146 was more pronounced in the tubular area (Fig. 5F). In db/db mice, the sCD146 level in serum samples was also much higher, and Box5 treatment had no obvious impact on the expression level of sCD146 (Fig. 5B).

JNK is a downstream factor of Wnt5a signaling, which can be altered by changes in diabetic status and is affected by Box5 treatment. Western blot analysis showed that JNK phosphorylation was increased in db/db mice, whereas Box5 treatment prevented the increased phosphorylation of JNK (Fig. 5C, F).

These data suggest that although the Wnt5a antagonist, Box5, had no significant effect on the expression levels of Wnt5a and CD146, it inhibited the activation of downstream JNK, which in turn suppressed the inflammatory process in db/db mice. This may work by blocking the binding of Wnt5a to its receptor (possibly CD146).

### Both Wnt5a antagonist treatment and Wnt5a knockdown prevented high glucose-induced inflammatory responses in HK-2 cells

We next explored the anti-inflammatory effect of Wnt5a inhibition in vitro. First, HK-2 cells were pretreated with Box5 (50, 100, or 500 μM/L) for 2 h, followed by treatment with high glucose (30 mM) for an additional 24 h. The results showed that HG stimulation significantly upregulated the mRNA expression of the proinflammatory cytokines TNF-α, IL-6, and CCL-2, while Box5 treatment prevented this upregulation in a dose-dependent manner. At a dose of 100 μM/L, Box5 treatment significantly inhibited the high glucose-induced inflammatory responses and the phosphorylation of JNK in HK-2 cells (Supplementary Fig. S3).

**Fig. 6:**
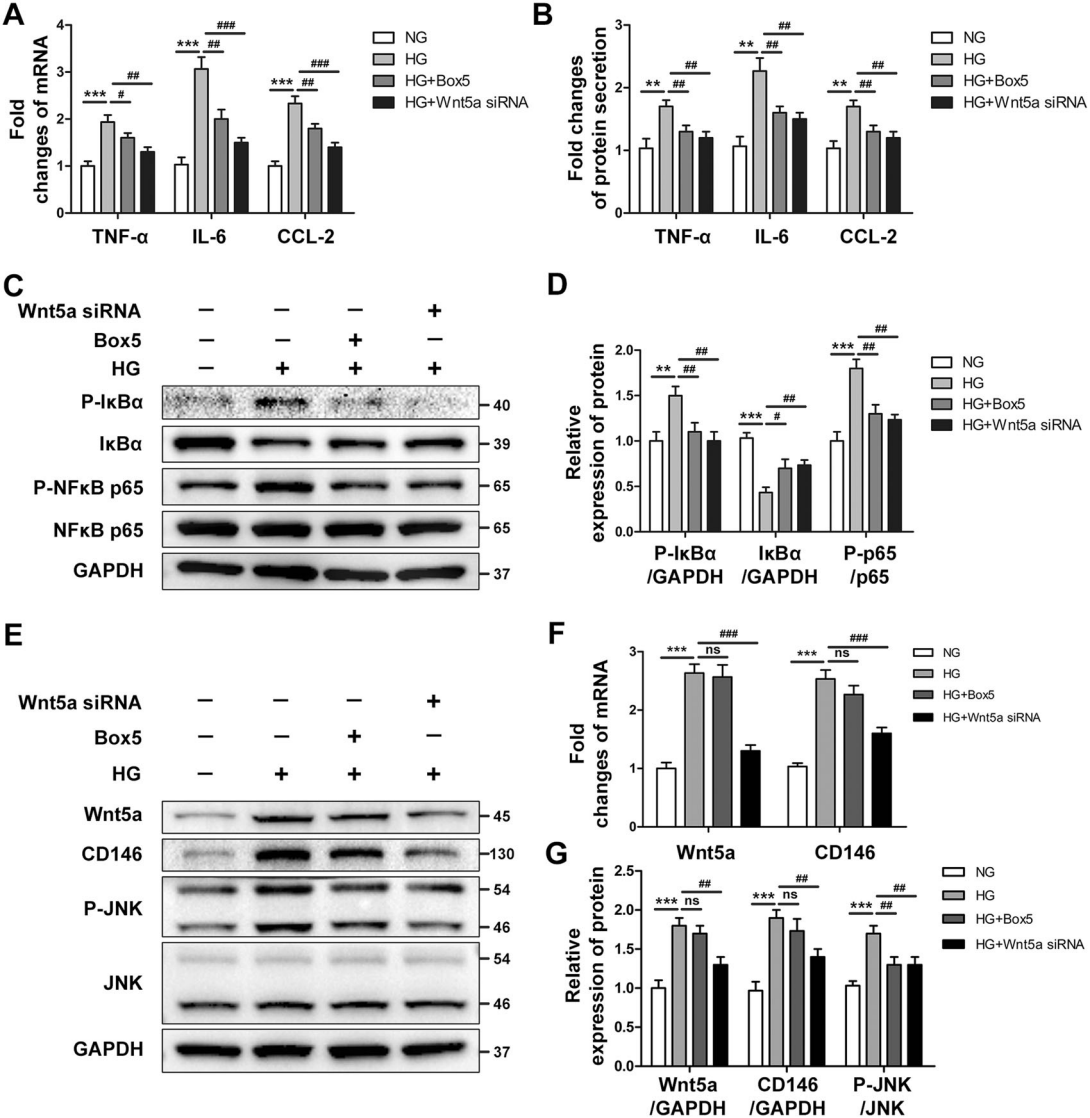
Both Wnt5a antagonist treatment and Wnt5a knockdown prevented high glucose-induced inflammatory responses in HK-2 cells. **A** HK-2 cells were transfected with 50 nM Wnt5a siRNA or pretreated with Box5 (100 μM/L) before exposure to high glucose (HG, 30 mM) for 24 h. The control group was incubated with normal glucose (NG, 5.5 mM). The mRNA expression of TNF-α, IL-6, and CCL-2 was determined by real-time PCR. ***P < 0.001 versus NG group, ^###^*P* < 0.001, ^##^*P* < 0.01, ^#^*P* < 0.05 versus HG group. **B** The protein synthesis of TNF-α, IL-6, and CCL-2 in culture supernatants was determined by ELISA. **P < 0.01 versus NG group, ^##^*P* < 0.01, versus HG group. **C**, **D** Representative western blot analysis and densitometric quantification of phosphorylated IκB, IκB, phosphorylated NF-κB p65, and NF-kB p65 in each group. ***P < 0.001, **P < 0.01 versus NG group, ^###^*P* < 0.001, ^##^*P* < 0.01, ^#^*P* < 0.05 versus HG group. **E**, **G** Representative western blot analysis and densitometric quantification of Wnt5a, CD146, phosphorylated JNK, and total JNK in each group. ***P < 0.001 versus NG group, ^##^*P* < 0.01 versus HG group, ns, no significance. **F** The mRNA levels of Wnt5a and CD146 were determined by real-time PCR in all groups. ***P < 0.001 versus NG group, ^###^*P* < 0.001 versus HG group, ns, no significance. PCR and ELISA experiments were performed in triplicate. Western blotting was performed in duplicate. All results were represented as the mean ± SD. The two-sided unpaired *t*-tests and one-way ANOVA followed by Bonferroni multiple comparison test were used.

We further validated the Wnt5a-mediated proinflammatory effect using gene-specific siRNA. HK-2 cells were transiently transfected with 50 nM Wnt5a-specific siRNA or pretreated with Box5 (100 μM/L) before exposure to HG. We found that Wnt5a siRNA and Box5 almost equally decreased HG-induced upregulation of TNF-α, IL-6, and CCL-2 gene expression and protein secretion (Fig. 6A, B). The increased phosphorylation of IκB and NF-κB p65 was also markedly reversed after Wnt5a knockdown or Box5 treatment (Fig. 6C, D). Although Box5 had no significant effect on the expression of Wnt5a and CD146, similar to our previous data in vivo, Wnt5a-specific siRNA markedly decreased the endogenous expression of Wnt5a and CD146. However, both Box5 treatment and Wnt5a knockdown inhibited the HG-induced activation of downstream JNK (Fig. 6E–G). In summary, both Wnt5a antagonist treatment and Wnt5a knockdown blocked the activation of noncanonical Wnt5a signaling and ameliorated high glucose-induced inflammation in HK-2 cells.

### CD146 was essential for Wnt5a-induced inflammation

**Fig. 7:**
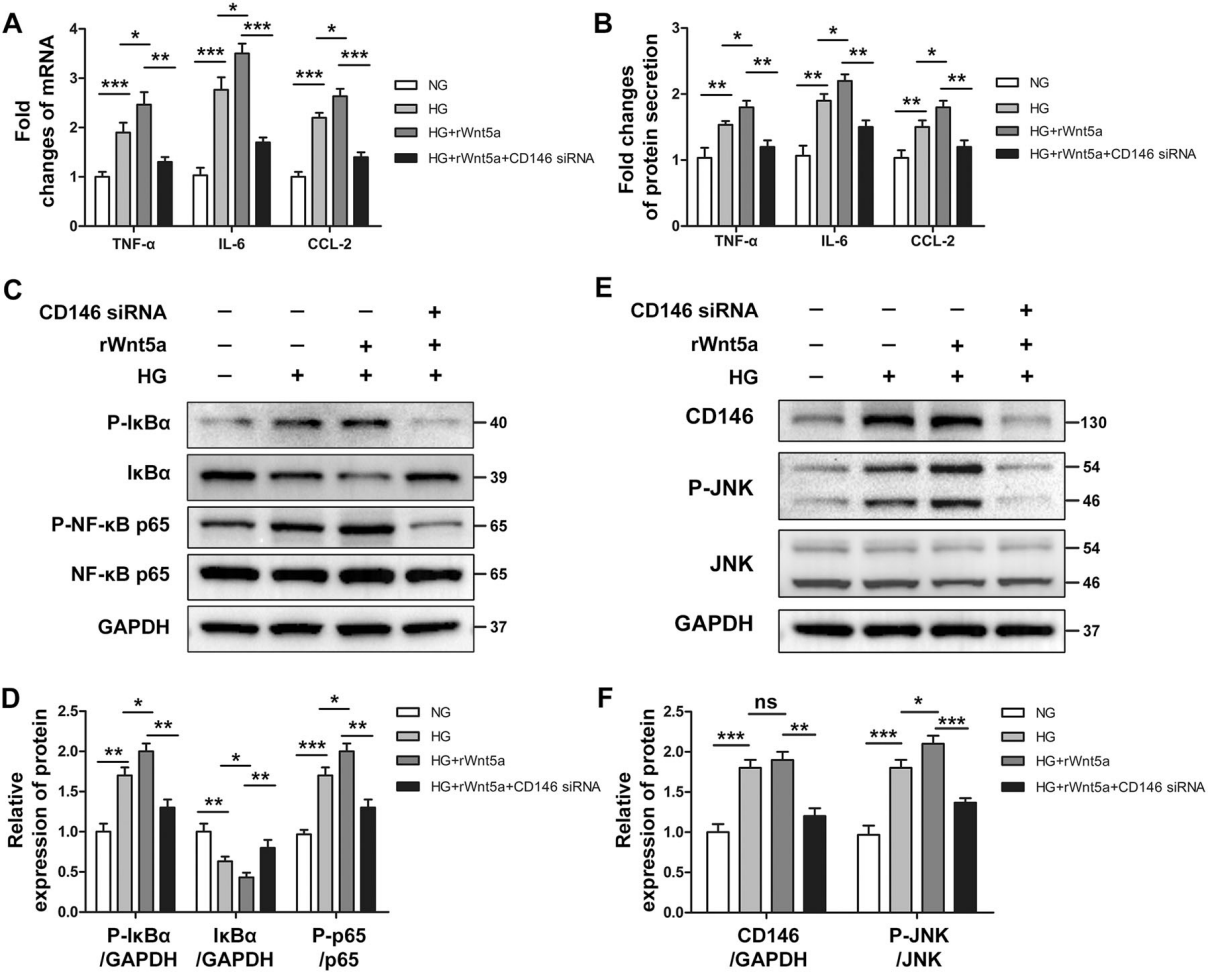
CD146 was essential for Wnt5a-induced inflammation. **A**, **B** HK-2 cells with no knockdown or transfected with CD146 siRNA were incubated with normal glucose (5.5 mM) or HG (30 mM) for 24 h in the absence or presence of recombinant Wnt5a (rWnt5a) protein (100 ng/mL, 4 h). The mRNA expression of TNF-α, IL-6, and CCL-2 was determined by real-time PCR. Protein synthesis of TNF-α, IL-6, and CCL-2 in culture supernatants was determined by ELISA. ***P < 0.001, **P < 0.01, *P < 0.05. **C**, **D** Representative western blot analysis and densitometric quantification of phosphorylated IκB, IκB, phosphorylated NF-κB p65, and NF-kB p65 in each group. ***P < 0.001, **P < 0.01, *P < 0.05. **E**, **F** Representative western blot analysis and densitometric quantification of CD146, phosphorylated JNK, and total JNK in each group. ***P < 0.001, **P < 0.01, *P < 0.05, ns, no significance. PCR and ELISA experiments were performed in triplicate. Western blotting was performed in duplicate. All results were represented as the mean ± SD. Two-sided unpaired *t*-tests and one-way ANOVA followed by Bonferroni multiple comparison test were used.

As Wnt5a/JNK signaling has been well studied previously, we next analyzed the role of CD146 in noncanonical Wnt signaling using CD146-specific siRNA. After exposure to HG for 20 h, HK-2 cells were incubated with recombinant Wnt5a protein (100 ng/mL) for another 4 h. The results showed that exogenous Wnt5a further increased HG-induced inflammatory responses (Fig. 7A–D). However, in HK-2 cells transfected with CD146-siRNA before exposure to HG and recombinant Wnt5a, the inflammatory responses and the phosphorylation of JNK were largely reduced (Fig. 7E, F). Thus, these data confirmed that CD146 was essential for Wnt5a-induced inflammation.

### Wnt5a interacted with CD146 directly in HK-2 cells

**Fig. 8:**
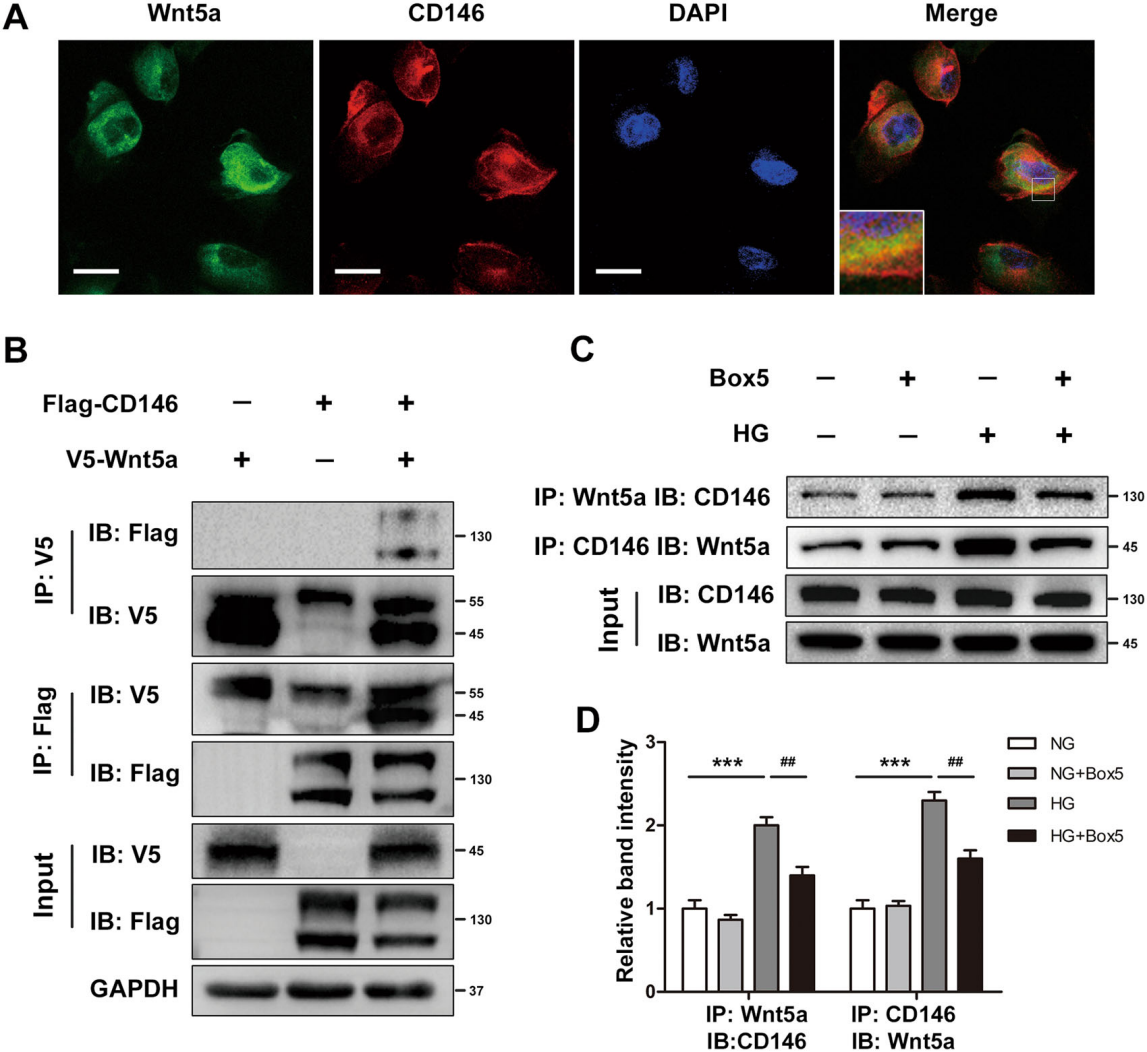
CD146 binds to Wnt5a directly in HK-2 cells. **A** Immunofluorescent double staining of Wnt5a and CD146 in HK-2 cells. Images were taken with a confocal microscope (original magnification ×1000, bar = 10 µm). **B** One microgram of V5-tagged Wnt5a and 1 μg of FLAG-tagged CD146 or 2 μg of each plasmid were transfected into HK-2 cells for 48 h. Cells were lysed and incubated with anti-V5 antibody or anti-FLAG antibody. The precipitated materials were used for western blot analysis using anti-FLAG and anti-V5 antibodies. **C**, **D** For endogenous coimmunoprecipitation (co-IP) experiments, HK-2 cells were stimulated with HG for 24 h with or without pretreatment with Box5 (100 μM/L). Cells were then lysed and incubated with anti-wnt5a or anti-CD146 antibodies for IP, followed by western blot analysis with anti-CD146 or anti-wnt5a antibodies. Input lysates were also assessed by western blot analysis with anti-wnt5a and anti-CD146 antibodies. ***P < 0.001 versus NG group, ^##^*P* < 0.01 versus HG group. The co-IP assay and immunostaining were performed in duplicate. All results were represented as the mean ± SD. Two-sided unpaired *t*-tests and one-way ANOVA followed by Bonferroni multiple comparison test were used.

A previous study indicated that Wnt5a uses CD146 as a receptor to regulate cell migration and zebrafish embryonic convergent extension. We then exposed the functional interaction between Wnt5a and CD146 in HK-2 cells. First, the immunofluorescent double staining demonstrated the colocalization of Wnt5a and CD146 in HK-2 cells (Fig. 8A). Then co-IP assays were performed to functionally verify whether CD146 binded to Wnt5a directly in HK-2 cells. We transiently transfected cells with V5-Wnt5a and Flag-CD146 plasmids. The results showed that V5-Wnt5a directly interacts with Flag-CD146 in cells coexpressing the two proteins (Fig. 8B). In addition, we confirmed the interaction of Wnt5a and CD146 by co-IP with endogenous proteins in HK-2 cells. HG promoted the expression and interaction of Wnt5a and CD146, while Box5 treatment inhibited the interaction of Wnt5a and CD146 without affecting the expression (Fig. 8C, D). Thus, all data suggest that CD146 is a receptor of Wnt5a and binds to Wnt5a to activate noncanonical Wnt signaling in HK-2 cells.

## Discussion

DN, a major microvascular complication of diabetes mellitus, is an important cause of end-stage renal disease. Despite increasing evidence indicating that immune and inflammatory factors play an important role in renal tubular damage in DN, the mechanisms are not fully understood^7,28^. In the current study, we validated for the first time that the expression of Wnt5a and CD146 is significantly upregulated in human DN. Through in vivo and in vitro experiments, we demonstrated that Wnt5a can directly bind to CD146 and activate JNK phosphorylation, thus triggering the subsequent inflammatory process and the progression of DN through the activation of noncanonical Wnt signaling.

Wnt5a signaling has recently been proposed to play an essential role in obesity- or diabetes-induced metabolic dysfunction and inflammation. In the adipose tissue of obese patients and mouse models, Wnt5a expression increases and promotes the expression of proinflammatory cytokines in adipocytes and macrophages^16,17^. Wnt5a also acts as an inflammatory mediator and contributes to impaired endothelial function in diabetes, atherosclerosis, and rheumatoid arthritis^18,19,29^. In the kidney, Han et al.^30^ found that the activation of the Wnt5a pathway promoted the epithelial–mesenchymal transition of human renal proximal tubule cells. However, the biological function of Wnt5a signaling in kidney injury, especially DN, remains incompletely understood. Here we demonstrated the upregulation of Wnt5a expression in human DN, which was found predominantly in the tubulointerstitial compartment compared with the glomerular compartment. In addition, quantitative analysis of the expression of Wnt5a staining demonstrated that the IOD value of Wnt5a staining was correlated with the interstitial infiltration of inflammatory cells, the expression of proinflammatory cytokines, IFTA, eGFR, and 24-h proteinuria. These data strongly suggest that Wnt5a signaling plays a role in the inflammatory process and the progression of human DN.

To confirm the role of Wnt5a in mediating tubulointerstitial inflammation and kidney injury, db/db mice were treated with a Wnt5a antagonist, Box5. Box5 is primarily used for cancer applications and it exerts its effect via directly binding to the receptor of Wnt5a, which inhibits the biological activity of Wnt5a signaling. Our results clearly showed that inhibiting Wnt5a by Box5 could ameliorate the kidney function and reduce the pathologic severity of DN without an obvious effect on the glucose level of db/db mice. Furthermore, this improvement was accompanied by a marked decrease in renal tubular CCL-2 expression and macrophage infiltration into the tubulointerstitium. These data indicated that the protective effect of Wnt5a inhibition on DN was due to the blockage of renal inflammation, which was independent of blood glucose levels.

Noncanonical Wnt5a signaling comprises a series of frequently overlapping pathways with two main branches: the Wnt5a/Ca^2+^ pathway and the Wnt5a/JNK or planar cell polarity (PCP) pathway^12,31^. Although studies^29^ showed that the Wnt5a/Ca^2+^ pathway regulated Wnt5a-induced inflammation in endothelial cells, no significant differences were found in the intracellular calcium level between HK-2 cells with and without transfection of V5-Wnt5a plasmids (Supplementary Fig. S4). Furthermore, JNK signaling has been widely demonstrated to be relevant in the setting of metabolic disease-induced inflammation^16,17^. Thus we hypothesized that the proinflammatory effects of Wnt5a in DN were mediated by activated JNK signaling. In this regard, we found that JNK phosphorylation was increased in db/db mice, whereas the Wnt5a antagonist prevented the increased activation of the JNK pathway. In cultured HK-2 cells, both Box5 treatment and Wnt5a knockdown inhibited the HG-induced phosphorylation of JNK and the expression of the proinflammatory cytokines TNF-α, IL-6, and CCL-2.

As for the activation of the Wnt5a/JNK pathway, it is well known that Wnt5a binds to the receptor Frizzled (Fz) and activates a scaffolding protein Disheveled (Dvl), and then activates small GTPase and JNK^31^. In addition to Fz, multiple molecules have been reported to function as coreceptors, including some transmembrane tyrosine kinases, such as Ror2^32,33^ and Ryk^34^, and several atypical adhesion molecules, such as Fat, Dachsous, and Flamingo^35^. CD146 is also a cell adhesion molecule, which was once identified as a noncanonical Wnt5a receptor in the regulation of cell motility and polarity during zebrafish gastrulation^26^. Previously, we reported that the expression of CD146 was correlated with diabetic renal injury^25^, but the explicit molecular mechanisms of CD146 deserve further investigation. Here we demonstrated that the expression of CD146 was upregulated in DN patients, db/db mice, and HG-induced HK-2 cells. Knockdown of CD146 blocked Wnt5a-induced expression of proinflammatory cytokines and activation of JNK, which suggests that CD146 is essential for the activation of Wnt5a pathway. Finally, we confirmed that Wnt5a interacted with CD146 directly to activate noncanonical Wnt signaling in HK-2 cells.

In addition to the expression of CD146 in kidney biopsy, a soluble form of CD146 (sCD146) has been identified to be expressed in the peripheral circulation of healthy or diseased subjects^36,37^. In DN patients, elevated circulating levels of sCD146 were confirmed in clinical studies^25,38^ and were correlated with disease severity. This suggests that this soluble biomarker may play a pivotal role in the pathogenesis of these diseases. In this study, we found that the increased concentration of sCD146 in serum and urine samples was related to tubulointerstitial inflammatory indicators and known prognostic factors in DN patients. This suggests that sCD146 may be a new potential biomarker for evaluating diabetic nephropathy in a convenient and noninvasive manner.

Taken together, our studies suggest that through binding to CD146, Wnt5a-induced noncanonical signaling is a contributing mechanism for renal tubular inflammation in diabetic nephropathy. The level of sCD146 in serum and urine is associated with kidney injury and could be a potential biomarker to predict renal outcomes in DN patients. Future studies are warranted to assess the therapeutic potential of Wnt5a-blocking strategies in diabetic kidney injury.

## Supplementary information

Supplementary Tables

Supplementary Figure legends

Supplementary Figure S1. Efficacy of Wnt5a and CD146 knockdown in HK-2 cells.

Supplementary Figure S2. No correlation was found between Wnt5a and CD146 expression and blood glucose and hemoglobin A1c (HbA1c) in DN patients.

Supplementary Figure S3. Wnt5a antagonist prevented high glucose induced inflammatory responses in HK-2 cells.

Supplementary Figure S4. The concentration of calcium in HK-2 cells with or without transfection of V5-Wnt5a plasmids.
