# Supplementary material for: Wnt5a promotes renal tubular inflammation in diabetic nephropathy by binding to CD146 through noncanonical Wnt signaling
Source: Cell Death Dis. 2021 Jan 18;12(1):92. doi: 10.1038/s41419-020-03377-x (PMC7814016; doi:10.1038/s41419-020-03377-x)
Supplement: Supplementary file 1 — Supplementary Tables [file 41419_2020_3377_MOESM1_ESM.docx]

**Supplementary Table S1 Primers used for Real-time PCR**

| **Human Primer** | | |
| --- | --- | --- |
| **Gene** | **Forward (5’ to 3’)** | **Reverse (5’ to 3’)** |
| hWnt5a | GCCAAGGAGTTCGTGGACGC | CAGCACATGAGCTCGCAGCC |
| hCD146 | AGAACCGGGTCCACATTCAG | GTCGGGTAGAAAACAGGGACG |
| hTNF-α | CGAGTGACAAGCCTGTAGC | GGTGTGGGTGAGGAGCACAT |
| hIL-6 | AAAGCAGCAAAGAGGCACTG | TACCTCAAACTCCAAAAGACCAG |
| hCCL-2 | CCTTCTGTGCCTGCTGCTC | GCTTCTTTGGGACACTTGCTG |
| hGAPDH | ATCCCATCACCATCTTCCAGG | GATGACCCTTTTGGCTCCC |
| **Mouse Primer** | | |
| **Gene** | **Forward (5’ to 3’)** | **Reverse (5’ to 3’)** |
| mWnt5a | CTGGCTCCTGTAGCCTCAAG | GCCGCGCTATCATACTTCTC |
| mCD146 | ATGTGGACAGGCAAGAGCTC | TGTACAAGCCACTGGACTCG |
| mTNF-α | CGGAGTCCGGGCAGG | GCTGGGTAGAGAATGGATGAA |
| mIL-6 | GCTACCAAACTGGATATAATCAGGA | CCAGGTAGCTATGGTACTCCAGAA |
| mCCL-2 | CAGCCAGATGCAGTTAACGC | GCCTACTCATTGGGATCATCTTG |
| mα-SMA | CCCTGAAGAGCATCCGACA | CTCCAGAGTCCAGCACAATACC |
| mCollagen-I | GAGGGCGAGTGCTGTGCT | GTCCAGGGATGCCATCTCG |
| mGAPDH | TGTTCCTACCCCCAATGTGTC | TGAAGTCGCAGGAGACAACC |

**Supplementary Table S2** **Western blot antibodies**

| **Antibody** | **Company** | **Cat.No** | **Dilution** |
| --- | --- | --- | --- |
| Wnt5a | Cell Signaling | # 2392S | 1:1000 |
| CD146 | Abcam | ab75769 | 1:1000 |
| p-JNK | Cell Signaling | # 4668S | 1:1000 |
| JNK | R&D | AF1387 | 1:1000 |
| p-p65 | Cell Signaling | #3033S | 1:1000 |
| P65 | Cell Signaling | #8242S | 1:1000 |
| GAPDH | Cell Signaling | #2118S | 1:5000 |
| β-actin | Thermo Fisher Scientific | AM4302 | 1:5000 |

**Supplementary Table S3. Clinical and pathological data of 15 patients with diabetic nephrology at biopsy**

| **Patients** | **Age(yr)**  **/Sex** | **Duration of DM(yr)** | **SCr**  **(μmol/L)** | **eGFR(ml/min per 1.73m^2^)** | **FBG**  **(mmol/L)** | **HbA1c**  **(%)** | **UPE/24h (g)** | **Pathologic classification of DN** | | | | |
| --- | --- | --- | --- | --- | --- | --- | --- | --- | --- | --- | --- | --- |
|  |  |  |  |  |  |  |  | **Glomerular classification** | **Percentage of IFTA (%)** | **Score**  **of interstitial inflammation** | **Score of arteriolar hyalinosis** | **Score of vessels arteriosclerosis** |
| 1 | 49/M | 18 | 74 | 108.6 | 12.26 | 13.7 | 2.98 | Ⅲ | 35 | 1 | 2 | 0 |
| 2 | 65/M | 15 | 85 | 86.78 | 7.73 | 8.5 | 3.45 | Ⅲ | 20 | 2 | 2 | 2 |
| 3 | 46/M | 3 | 63 | 111.76 | 8.66 | 7.7 | 2.07 | Ⅲ | 20 | 1 | 2 | 2 |
| 4 | 58/F | 20 | 157 | 27.74 | 19.34 | 10.9 | 9.26 | Ⅲ | 40 | 1 | 2 | 1 |
| 5 | 54/F | 1 | 39 | 185.82 | 8.18 | 10.5 | 0.87 | IIa | 20 | 1 | 2 | 2 |
| 6 | 55/M | 20 | 111 | 64.29 | 8.51 | 12.3 | 1.9 | IIa | 35 | 1 | 2 | 1 |
| 7 | 55/M | 6 | 179 | 35.77 | 9.55 | 17.9 | 7.36 | Ⅳ | 45 | 2 | 2 | 1 |
| 8 | 57/M | 10 | 217 | 28.02 | 4.46 | 7.5 | 6.08 | Ⅲ | 60 | 1 | 2 | 1 |
| 9 | 61/M | 9 | 86 | 86.01 | 4.93 | 9.2 | 2.46 | IIb | 30 | 1 | 2 | 0 |
| 10 | 49/M | 14 | 113 | 64.18 | 8.44 | 9.7 | 3.3 | Ⅲ | 35 | 1 | 2 | 1 |
| 11 | 42/M | 12 | 89 | 88.9 | 4.85 | 11.9 | 2.85 | Ⅲ | 35 | 1 | 2 | 1 |
| 12 | 67/M | 2 | 53 | 155.0 | 10.39 | 8.2 | 0.74 | IIa | 15 | 1 | 1 | 1 |
| 13 | 63/F | 21 | 121 | 44.58 | 3.42 | 7.9 | 4.16 | Ⅲ | 50 | 1 | 2 | 1 |
| 14 | 64/M | 30 | 132 | 44.76 | 12.09 | 4.7 | 4.1 | Ⅳ | 55 | 1 | 2 | 1 |
| 15 | 47/M | 0.5 | 215 | 37.04 | 6.44 | 7.5 | 3.57 | IIb | 40 | 2 | 2 | 1 |
| mean±SD | 55.5±7.7 | 12.1±8.7 | 115.6±55.4 | 78.0±47.0 | 8.6±4.0 | 9.9±3.2 | 3.7±2.3 |  |  |  |  |  |
