# Supplementary material for: Wnt5a promotes renal tubular inflammation in diabetic nephropathy by binding to CD146 through noncanonical Wnt signaling
Source: Cell Death Dis. 2021 Jan 18;12(1):92. doi: 10.1038/s41419-020-03377-x (PMC7814016; doi:10.1038/s41419-020-03377-x)
Supplement: Supplementary file 2 — Supplementary Figure legends [file 41419_2020_3377_MOESM2_ESM.docx]

**Supplementary Figure S1. Efficacy of Wnt5a and CD146 knockdown in HK-2 cells.**

(A), (B), (C) Efficacy of Wnt5a knockdown in HK-2 cells. HK-2 cells were transfected with 50nM nonspecific negative control siRNA (NC siRNA) or Wnt5a-specific siRNA (Wnt5a siRNA), and the respective mRNA and protein expressions were measured after 48-72 hours. ***P<0.001, **P<0.01. (D), (E), (F) Efficacy of CD146 knockdown in HK-2 cells. HK-2 cells were transfected with 2 μg nonspecific negative control siRNA (NC siRNA) or CD146-specific siRNA (CD146 siRNA), and the respective mRNA and protein expressions were measured after 48-72 hours. ***P<0.001. PCR were performed in triplicate. Western blot were performed in duplicate. All results were represented as means± SD. The two-sided unpaired t-test and oneway ANOVA followed by Bonferroni multiple comparison test were used.

**Supplementary Figure S2. No correlation was found between Wnt5a and CD146 expression and blood glucose and hemoglobin A1c (HbA1c) in DN patients.**

Correlation between the intensity of Wnt5a staining, CD146 staining and the concentration of sCD146 in serum and urine samples, and blood glucose and HbA1c was calculated in all DN patients using Pearson and Spearman correlation analysis. P and R^2^ were indicated on the graph.

**Supplementary Figure S3. Wnt5a** **antagonist prevented high glucose induced inflammatory responses in HK-2 cells.**

(A), (B), (C) HK-2 cells were pretreated with Box5 (50, 100, or 500 μM/L) for 2 hours, followed by treatment with high glucose (30 mM) for an additional 24 hours. The mRNA levels of proinflammatory cytokines TNF-α, IL-6 and CCL-2/MCP-1 was determined by real-time PCR in all groups. ***P<0.001, versus NG, ^###^P<0.001, ^##^P<0.01, ^#^P<0.05, versus HG. (D) Representative Western blot analysis of the phosphorylated JNK and total JNK in all groups. (E) Quantification of Western blot by densitometric analysis. ***P<0.001, versus NG, ^##^P<0.01, ^#^P<0.05, versus HG, ns, no significance. PCR experiments were performed in triplicate. Western blot were performed in duplicate. All values were presented as means±SD. The two-sided unpaired t-test and oneway ANOVA followed by Bonferroni multiple comparison test were used. NG, normal glucose; HG, high glucose.

**Supplementary Figure S4. The concentration of calcium in HK-2 cells with or without transfection of V5-Wnt5a plasmids.**

HK-2 cells were transiently transfected with or without 2μg V5-Wnt5a plasmids using Lipofectamine 3000 (Invitrogen) according to the manufacturer’s protocol. The intracellular calcium levels were measured by the fluorescent calcium indicator, Fluo4. Original magnification, ×400; scale bar, 200 μm.
