# Supplementary figures and images for: Wnt5a promotes renal tubular inflammation in diabetic nephropathy by binding to CD146 through noncanonical Wnt signaling
Source: Cell Death Dis. 2021 Jan 18;12(1):92. doi: 10.1038/s41419-020-03377-x (PMC7814016; doi:10.1038/s41419-020-03377-x)

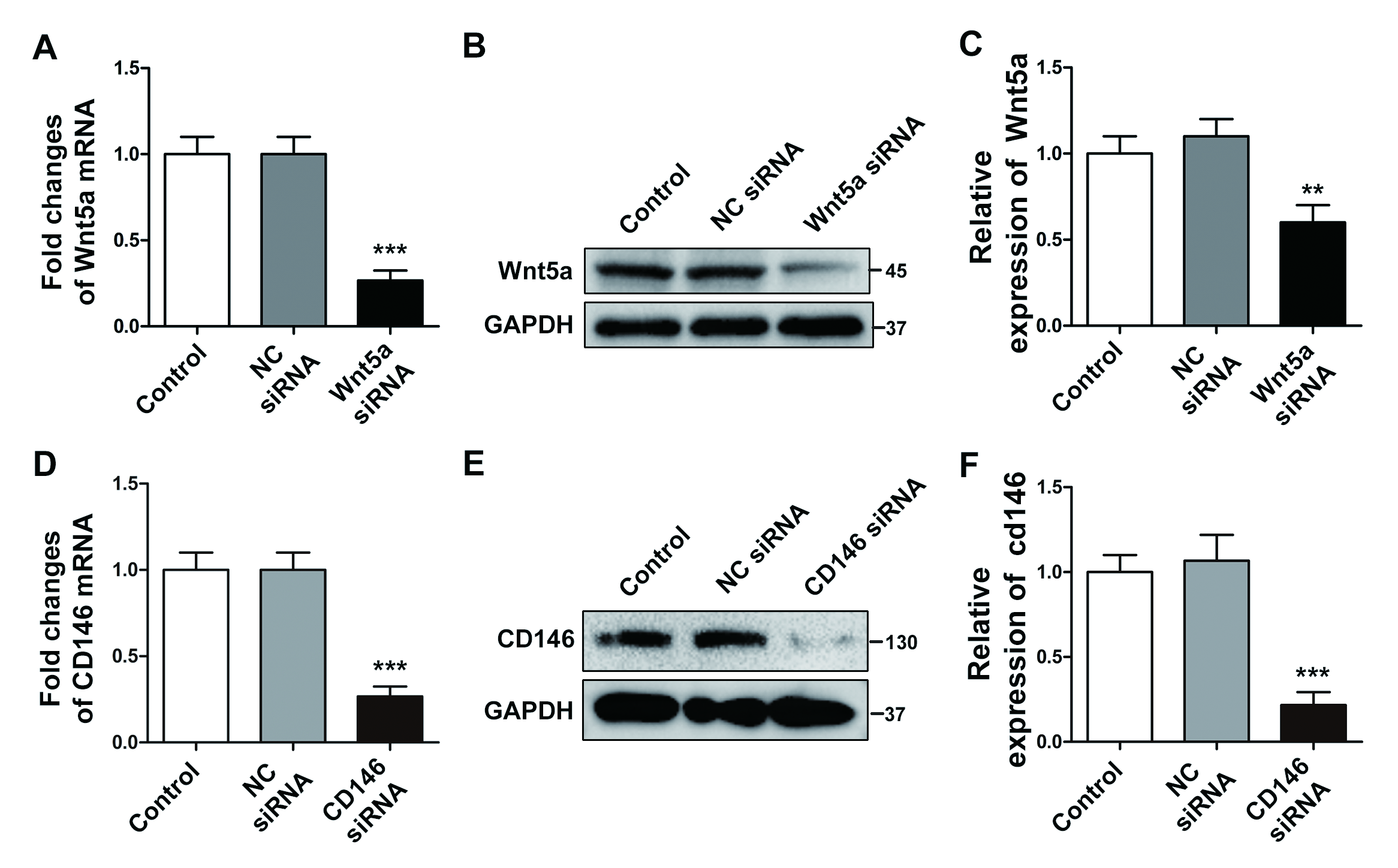

Supplement: Supplementary file 3 — Supplementary Figure S1. Efficacy of Wnt5a and CD146 knockdown in HK-2 cells. [file 41419_2020_3377_MOESM3_ESM.tif]

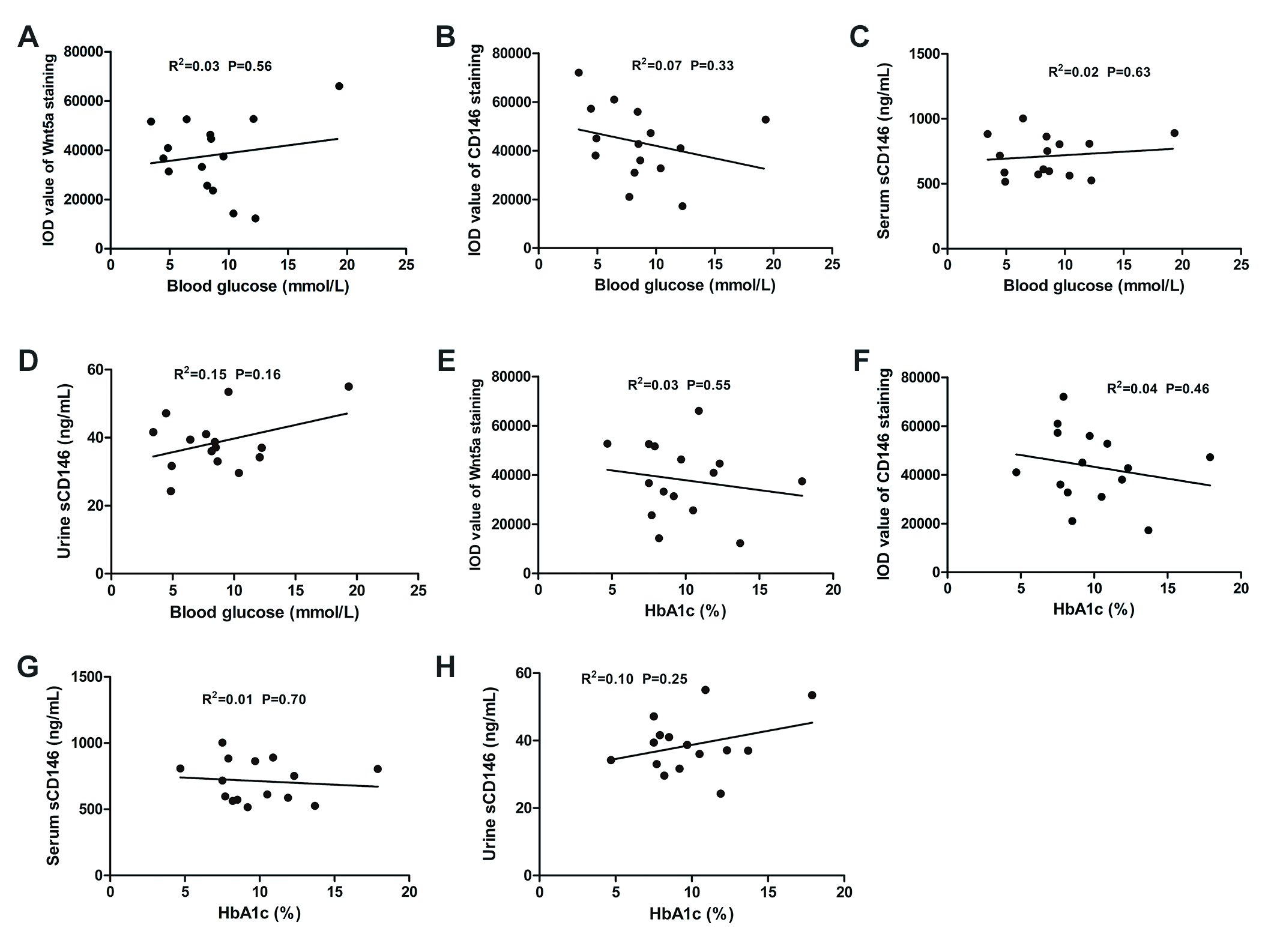

Supplement: Supplementary file 4 — Supplementary Figure S2. No correlation was found between Wnt5a and CD146 expression and blood glucose and hemoglobin A1c (HbA1c) in DN patients. [file 41419_2020_3377_MOESM4_ESM.tif]

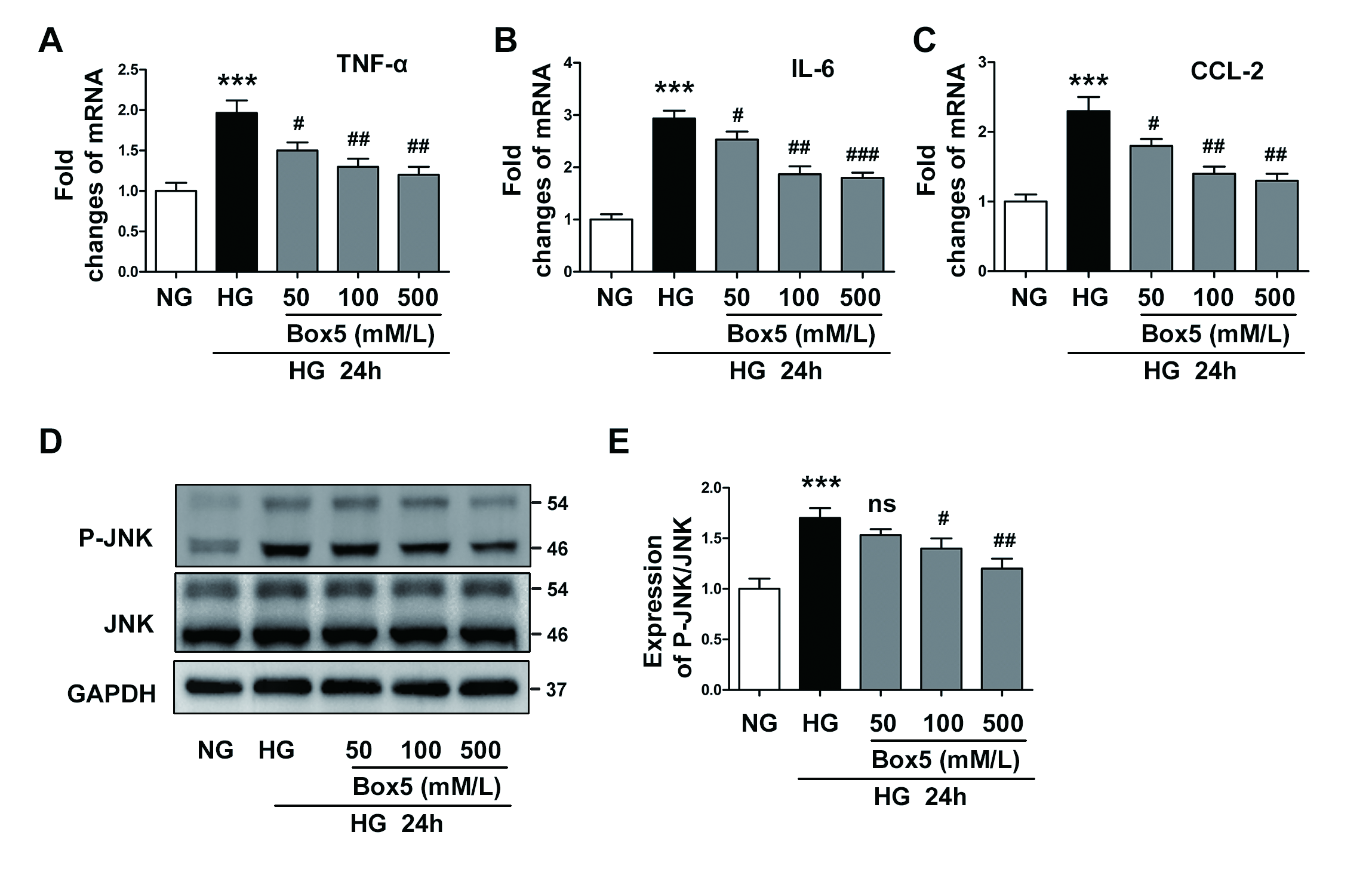

Supplement: Supplementary file 5 — Supplementary Figure S3. Wnt5a antagonist prevented high glucose induced inflammatory responses in HK-2 cells. [file 41419_2020_3377_MOESM5_ESM.tif]

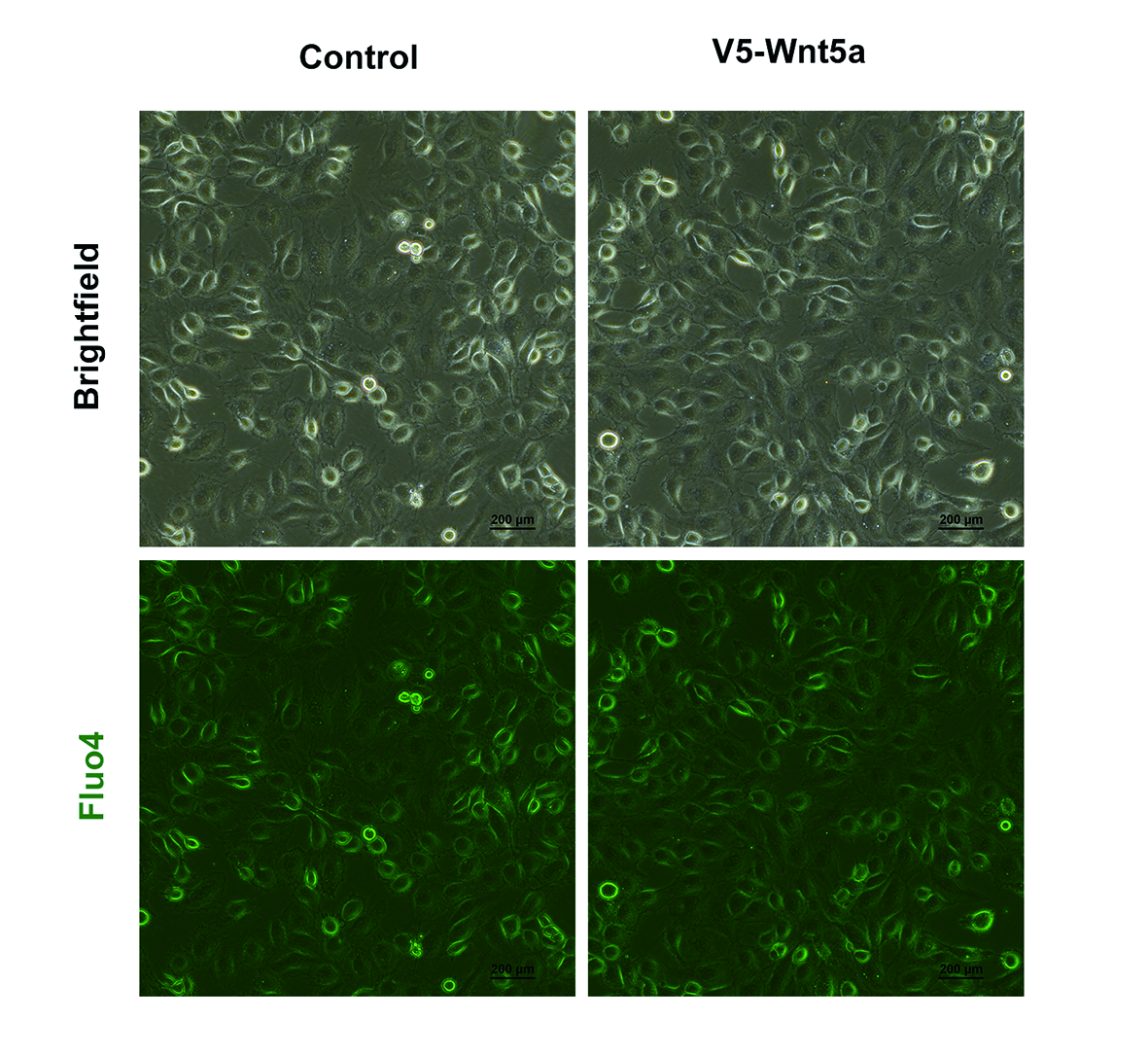

Supplement: Supplementary file 6 — Supplementary Figure S4. The concentration of calcium in HK-2 cells with or without transfection of V5-Wnt5a plasmids. [file 41419_2020_3377_MOESM6_ESM.tif]
